# Metabolic tumour volume on ^18^F-FDG PET/CT predicts extended pathological T stages in patients with renal cell carcinoma at staging

**DOI:** 10.1038/s41598-021-03023-2

**Published:** 2021-12-06

**Authors:** Dongwoo Kim, Narae Lee, Suk Hyun Lee, Hyun Jeong Kim, Hye-Suk Hong, Jee Soo Park, Nam-Hoon Cho, Young Deuk Choi, Won Sik Ham, Seung Hwan Lee, Woong Kyu Han, Mijin Yun

**Affiliations:** 1 Department of Nuclear Medicine, Severance Hospital, Yonsei University College of Medicine, 50-1 Yonsei-ro, Seodaemun-gu, Seoul, 03722 Republic of Korea; 2 Department of Nuclear Medicine, Wonju Severance Christian Hospital, Yonsei University Wonju College of Medicine, 20 Ilsan-ro, Wonju, 26426 Republic of Korea; 3 Department of Radiology, Kangnam Sacred Heart Hospital, Hallym University, Seoul, Republic of Korea; 4 Department of Nuclear Medicine, Yongin Severance Hospital, Yonsei University College of Medicine, Seoul, Gyeonggi-do Republic of Korea; 5 Department of Diagnostic Radiology, National Cancer Center, Goyang, Republic of Korea; 6 Department of Urology, Severance Hospital, Yonsei University College of Medicine, 50-1 Yonsei-ro, Seodaemun-gu, Seoul, 03722 Republic of Korea; 7 Department of Pathology, Severance Hospital, Yonsei University College of Medicine, 50-1 Yonsei-ro, Seodaemun-gu, Seoul, Republic of Korea

**Keywords:** Cancer, Oncology, Urology

## Abstract

We evaluated the predictive value of ^18^F-fluorodeoxyglucose (FDG) uptake on positron emission tomography/CT (PET/CT) for extended pathological T (pT) stages (≥ pT3a) in Renal cell carcinoma (RCC) patients at staging. Thirty-eight RCC patients who underwent ^18^F-FDG PET/CT at staging, followed by radical nephrectomy between September 2016 and September 2018, were included in this prospective study. Patients were classified into two groups (limited pT stage: stage T1/2, n = 17; extended pT stage: T3/4, n = 21). Univariate and multivariate logistic regression analyses were performed to identify clinicopathological and metabolic variables to predict extended pT stages. ^18^F-FDG metabolic parameters were compared in relation to International Society of Urological Pathology (ISUP) grade and lymphovascular invasion (LVI). In univariate analysis, maximum standardised uptake value, metabolic tumour volume (MTV), and ISUP grade were significant. In multivariate analysis, MTV was the only significant factor of extended pT stages. With a cut-off MTV of 21.2, an area under the curve was 0.944, which was higher than 0.824 for clinical T stages (p = 0.037). In addition, high MTV, but not tumour size, was significantly correlated with aggressive pathologic features (ISUP grade and LVI). High glycolytic tumour volume on ^18^F-FDG PET/CT in RCC patients at staging is predictive of extended pT stages which could aid decision-making regarding the best type of surgery.

## Introduction

Renal cell carcinomas (RCCs) are the seventh leading cause of cancer and accounts for approximately 90% of all renal tumours and 3.8% of all new malignancies^1^. Advances in imaging techniques have contributed to the increased incidence of localised RCCs and an improved 5-year survival rate. Of the types of surgical resection used for treating RCCs, radical nephrectomy (open or laparoscopic) is reserved for T3–4 diseases, while partial nephrectomy is considered in T1–2 diseases^2–4^. However, in some patients with localised RCC, partial nephrectomy may not be suitable because of locally extended tumour growth or unfavourable tumour location. Therefore, accurate preoperative staging of RCCs is vital for appropriate treatment decision-making.

Although contrast-enhanced CT of the abdomen is the diagnostic modality of choice for staging primary RCC, it has significant limitations in diagnosing extended pathological T (pT) stages. In predicting pT4 disease, the positive predictive value of CT is insufficient because of the difficulties in distinguishing abutment from direct invasion^5^. For the same reasons, accurate assessment of renal sinus fat invasion (pT3a) and perinephric tumour extension (pT3a) is also challenging on CT^6–9^. Therefore, there is a need for advanced imaging techniques to predict local pT staging for facilitating better surgical decision-making. ^18^F-fluorodeoxyglucose (FDG) uptake on positron emission tomography/CT (PET/CT) has been shown to be useful in determining the extent of surgery required in patients with hepatocellular carcinomas (HCCs)^20^. Moreover, overall survival and recurrence-free survival were significantly better following major hepatectomy than minor hepatectomy in patients in whom the preoperative maximum standardised uptake value (SUV_max_) of the tumour was ≥ 4.

Unlike most malignancies that have Warburg’s aerobic glycolysis, there is a significant proportion of RCCs without Warburg’s metabolic phenotype. In addition, the accumulation of excreted high ^18^F-FDG uptake in the pelvo-caliceal systems masks the detection of small tumours and is a major problem when using ^18^F-FDG PET/CT in RCC evaluation^11–13^. Accordingly, current NCCN clinical practice guidelines do not recommend FDG PET/CT alone to diagnose RCCs or follow-up for evidence of recurrence after surgery^1^. Nevertheless, several previous studies have reported the usefulness of ^18^F-FDG uptake in RCCs for predicting aggressive tumour features such as high International Society of Urological Pathology (ISUP) grade, distant metastases, and poor patient survival^11,13–17^. From a practical point of view, primary tumour detection may be beyond the scope of ^18^F-FDG PET/CT in RCCs; however, it could assist with the biological characterisation of RCCs based on the metabolic reprogramming found in individual RCCs.

The purpose of this prospective study was to evaluate the predictive values of the degree and volume of glycolysis on ^18^F-FDG PET/CT for extended T stages (≥ pT3a) in patients with RCCs at staging.

## Patients and methods

### Study subjects

Thirty-eight patients with RCCs who underwent ^18^F-FDG PET/CT at staging, followed by radical nephrectomy between September 2016 and September 2018, were included in this prospective study. The inclusion criteria were an RCC > 7 cm and either abutment or extension into vessels, sinus fat, perirenal fat, the pelvo-caliceal system, or adjacent organs based on abdominal CT. The interval between ^18^F-FDG PET/CT scans and surgery was 1–14 days with a mean of 1.6 ± 3.2 days. No treatment was performed before surgery in any of the patients. Clinical parameters, such as sex and age, and histopathological features, including tumour size, histological subtype, ISUP grade, lymphovascular invasion (LVI), and pathological staging, were assessed. Pathological tumour staging was performed according to the 8th TNM criteria proposed by the American Joint Committee on Cancer (AJCC) in 2017^18^. This prospective study was approved by the Institutional Review Board of the Yonsei University Health System (project no: 1-2016-0032), and informed consent was obtained from all patients. This study was registered at cris.nih.go.kr (KCT0006218).

### PET/CT imaging protocol

All patients fasted for at least 6 h before ^18^F-FDG PET/CT. Blood glucose levels of ≤ 140 mg/dL were ensured before the injection of 3.7 MBq of ^18^F-FDG per kg of body weight. PET/CT images were acquired using a PET/CT scanner (Discovery 710; General Electric Medical Systems, Milwaukee, WI, USA) with a low-dose CT transmission scan (tube voltage, 120 kV; tube current, auto mA). PET emission scans were acquired from the cerebellum to the proximal thighs for 2 min per bed position in three-dimensional mode. The acquired images were reconstructed using an iterative reconstruction algorithm (VUE point FX-SharpIR: iteration, 2; subset, 16; filter cut-off, 5 mm) and CT images were subjected to attenuation correction.

### Image analysis

^18^F-FDG PET/CT images were registered on contrast-enhanced CT or MR images using the imaging software MIM (MIM-6.5; MIM Software Inc., Cleveland, OH, USA). On each slice of the axial fusion images, we drew a region of interest (ROI) on the boundary of the tumour seen on contrast-enhanced CT or MRI, and erased the areas other than tumour to avoid excretory activity in the renal calyces, pelvis, and upper ureters. Then, all the ROIs were combined to create a VOI to measure the standardised uptake value (SUV) as follows:

$$\frac{\mathrm{decay}-\mathrm{corrected \,\, activity }\,\, [\mathrm{kBq}]/\mathrm{tissue\,\,  volume }\,\, [\mathrm{mL}]}{\mathrm{injected\,\,  }18\mathrm{F}-\mathrm{FDG \,\, activity }\,\, [\mathrm{kBq}]/\mathrm{body\,\,  mass }\,\, [\mathrm{g}]}$$

The SUVmax was defined as the highest pixel value of SUV within a VOI. The metabolic tumour volume (MTV) was defined as the total tumour volume above the SUV cut-off corresponding to the 97.5th percentile value in the contralateral kidney ([mean SUV of the contralateral kidney] + [two standard deviations of mean SUV in the contralateral kidney]) in the VOI.

### Statistical analysis

For statistical analyses, patients were classified into two groups according to pT stages: a limited group with pT1/2 stages (*n* = 17) and an extended group with pT3/4 stages (*n* = 21). For ISUP grades, grades 1 and 2 were considered low-grade, and grades 3 and 4 were considered high-grade. Clinical T (cT) staging was based on image analysis of unenhanced, nephrographic, or portal-venous phases on CT. Tumours limited to the kidney were categorised as T1 or T2 based on their size. T3 tumours were those with significant abutment or gross extension into the major veins, sinus fat, or perinephric tissues. Tumours with direct extension to the ipsilateral adrenal gland or invasion beyond Gerota’s fascia were classified as T4. Patient characteristics were compared between the limited and extended groups using a chi-square or Fisher’s exact test for categorical data. A one-way analysis of variance or Kruskal–Wallis test was used for continuous data. In addition, Pearson’s correlation analyses were performed to determine the relationship between metabolic features on PET/CT, ISUP grade, and tumour size.

Univariate and multivariate logistic regression analyses were used to predict the extended group with pT3/4 stages. The variables for logistic analyses were sex, age at diagnosis, tumour size, and metabolic parameters (SUV_max_ and MTV) on ^18^F-FDG PET/CT, and ISUP grade. We did not include independent variables, including zero values, in our analyses. A receiver operating characteristic (ROC) curve analysis was performed to determine the optimal cut-off for each significant predictor associated with the extended group. The sensitivity, specificity, and positive (PPV) and negative predictive values (NPV) for predicting the extended group were derived separately for different predictors using cross-tabulations. Statistical analyses were performed using SPSS (version 25.0; IBM, Armonk, NY, USA). For all tests, two-sided p-values < 0.05 were considered statistically significant.

### Ethics approval

All procedures involving human participants were performed in accordance with the ethical standards of the institutional and/or national research committee and with the 1964 Helsinki Declaration and its later amendments or comparable ethical standards.

### Consent to participate

Written informed consent was obtained from all study participants.

## Results

### Patient characteristics according to pT stages

Of 38 patients, 20 (52.6%) were men and 18 (47.4%) were women with a mean age of 60.7 ± 12.7 years and a mean tumour size of 8.2 ± 2.3 cm. There were 17 patients in the limited group (pT1/2) and 21 patients in the extended group. ISUP grades were low (grades 1 and 2) in 12 patients and high (grades 3 and 4) in 26 patients. Thirty-two tumours (84.2%) were of clear cell type, four (10.5%) were chromophobe, and two (5.3%) were of papillary type. LVI was present in 11 patients (28.9%). The clinicopathological characteristics and metabolic parameters of ^18^F-FDG PET/CT are summarised in Table 1.

**Table 1:** Patient clinical characteristics.

Characteristics	Limited T stage group; T1–2 (n = 17)	Extended T stage group; T3–4 (n = 21)	P-value
**Sex**			
Men, n (%)	7 (41.2%)	13 (61.9%)	0.328
Women, n (%)	10 (58.8%)	8 (38.1%)	
Age (years, mean ± SD)	57.8 ± 14.3	63.1 ± 11.1	0.325
Tumor size (cm, mean ± SD)	7.7 ± 2.1	8.6 ± 2.4	0.179
**Clinical T stage**			
T1, n (%)	3 (17.6%)	0 (0%)	0.001
T2, n (%)	8 (47.1%)	0 (0%)	
T3, n (%)	6 (35.3%)	17 (81.0%)	
T4, n (%)	0 (0%)	4 (19.0%)	
**Histopathology**			
Clear cell type, n (%)	13 (76.5%)	19 (90.5%)	0.423
Chromophobe type, n (%)	3 (17.6%)	1 (4.8%)	
Papillary type, n (%)	1 (5.9%)	1 (4.8%)	
^**18**^**F-FDG PET**			
SUVmax (mean ± SD)	4.5 ± 2.2	10.5 ± 6.3	< 0.001
MTV (mean ± SD)	13.4 ± 30.2	132.2 ± 92.9	< 0.001
**ISUP grade**			
Grade 1–2, n (%)	11 (64.7%)	1 (4.8%)	< 0.001
Grade 3–4, n (%)	6 (35.3%)	20 (95.2%)	
**Lymphovascular invasion**			
Yes	0 (0%)	11 (52.4%)	< 0.001
No	17 (100.0%)	10 (47.6%)	

**Figure 1:**
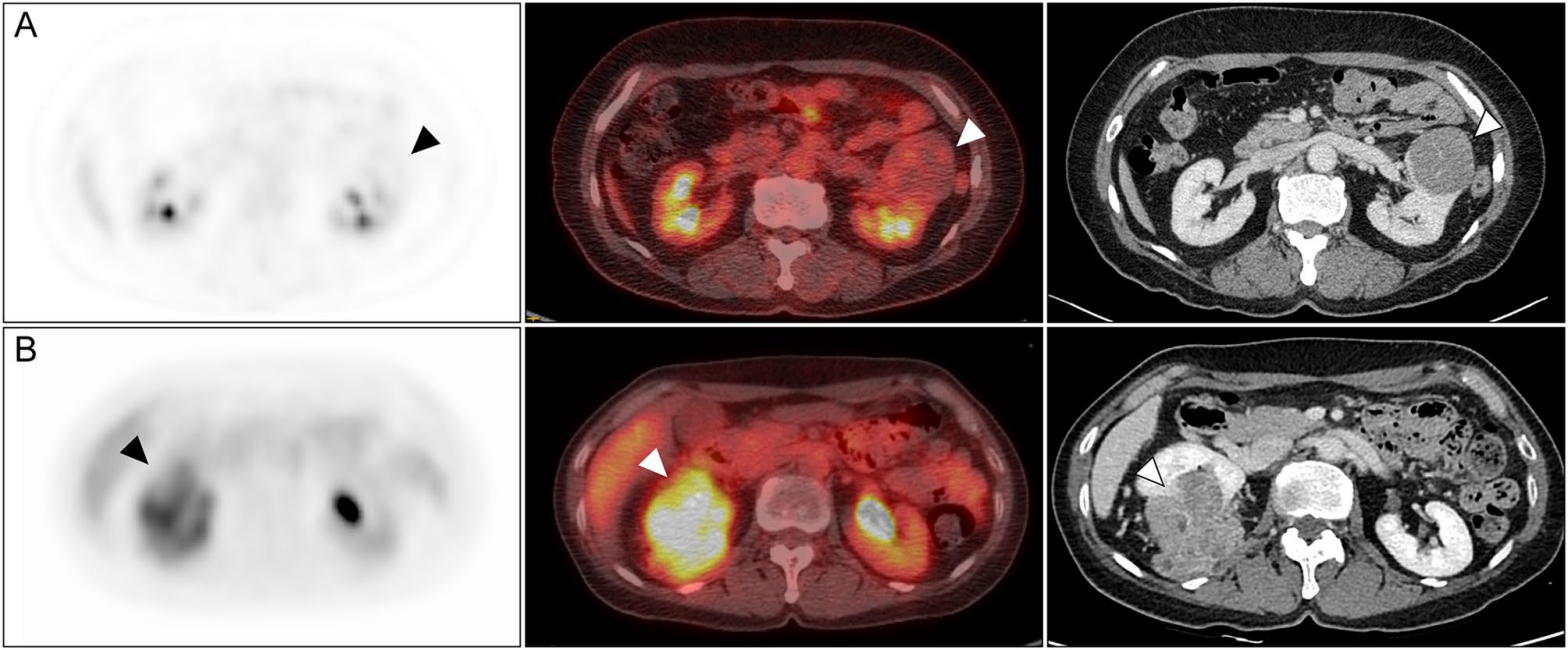
RCC with low and high MTV. Upper row: a 66-year-old female had a renal mass (arrowhead) with low ^18^F-FDG uptake (SUV_max_: 2.98, MTV: 1.44 cm^3^). The pathological T stage was T1b. Lower row: a 63-year-old female had a renal mass (arrowhead) with high ^18^F-FDG uptake (SUV_max_: 6.85, MTV: 99.16 cm^3^). The pathological T stage was T3a. *RCC* renal cell carcinoma, *FDG* fluorodeoxyglucose, *SUV*_*max*_ maximum standardised uptake value, *MTV* metabolic tumour volume.

**Figure 2:**
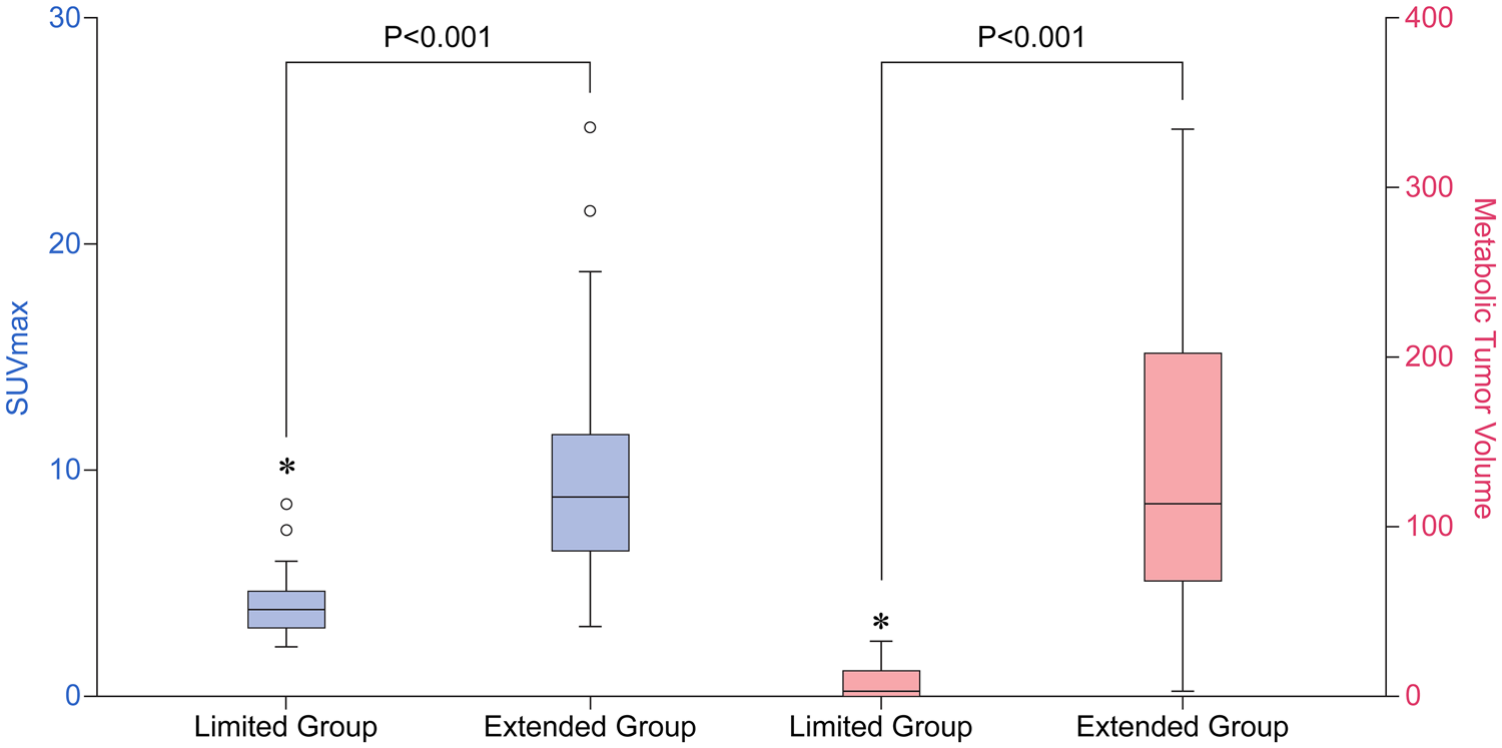
SUV_max_ and metabolic tumour volume differed significantly between limited and extended groups (circles outliers, > 1.5 times interquartile range; asterisks extreme outliers, > 3 times interquartile range).

Between the limited and extended groups, statistically significant differences were demonstrated in cT stage, SUV_max_, MTV, and ISUP grade. For cT stage on CT, tumours of 6/17 (35.3%) patients were over-staged as cT3 in the limited group, whereas those in 3/21 (14%) patients were misclassified as cT4 in the extended group. In terms of ISUP grades, 11/17 (64.7%) patients in the limited group had low-grade tumours, whereas 20/21 (95.2%) in the extended group had high-grade tumours (p = 0.001). The SUV_max_ of the primary RCCs were 4.5 ± 2.2 in the limited group and 10.5 ± 6.3 in the extended group (p < 0.001), while the MTV was 13.4 ± 30.2 and 132.2 ± 92.9, respectively (p < 0.001) (Figs. 1, 2). Importantly, there was no significant difference in tumour size between the limited and extended groups (7.7 ± 2.1 vs. 8.6 ± 2.4, respectively; p = 0.179).

### Tumour size versus ^18^F-FDG metabolic features according to ISUP grade and LVI

We found no significant correlation between tumour size and SUV_max_ on PET/CT, but a mild correlation between tumour size and MTV (r = 0.031, p = 0.86 for tumour size and SUV_max_ and r = 0.383, p = 0.018 for tumour size and MTV) was noted. In addition, no significant correlation was found between tumour size and ISUP grade (7.85 ± 2.06 vs 8.32 ± 2.40, p = 0.563). Unlike tumour size, higher SUV_max_ and MTV were noted in high-grade RCCs than in low-grade tumours (4.07 ± 2.09 vs. 9.52 ± 6.05, p = 0.005 for SUV_max_ and 12.90 ± 35.64 vs. 109.60 ± 95.79, p = 0.002 for MTV, respectively). Next, we evaluated tumour size, SUV_max_, and MTV with regard to the presence of LVI. Both tumour size and SUV_max_ showed no significant differences according to the presence of LVI (8.08 ± 2.36 vs. 8.39 ± 2.15, p = 0.710 for tumour size and 6.95 ± 5.59 vs. 9.87 ± 5.73, p = 0.156 for SUV_max_). However, a significantly higher MTV on ^18^F-FDG PET/CT was noted in the presence of LVI than in the absence of LVI (49.99 ± 79.99% vs. 150.45 ± 86.54, p = 0.002). Taken together, MTV on PET/CT appeared to be associated with aggressive pathological features, such as high grade of ISUP grade and LVI.

### Logistic regression analyses for the prediction of extended pT stages

In the univariate logistic regression analysis, SUV_max_ (p = 0.006), MTV (p = 0.005) on ^18^F-FDG PET/CT and ISUP grade (p = 0.002) were significant factors for predicting extended pT stages. Although tumour size is an important component of current TNM staging for RCCs in the limited group, it was insignificant for predicting the extended group. In the multivariate analysis, MTV (p = 0.032) was the only significant factor for predicting extended pT stages (Table 2). An ROC analysis with a cut-off MTV of 21.2 showed an area under the curve (AUC) of 0.944 (95% CI, 0.818–0.992, p < 0.001). The sensitivity, specificity, and PPV, NPV and accuracy of MTV (> 21.2) were 95.2%, 88.2%, 90.9%, 93.8%, and 92.1% respectively. There was a statistically significant difference in the AUCs between MTV and cT stages (0.944 for MTV vs. 0.824 for cT stage, p = 0.037).

**Table 2:** Univariate and multivariate analyses for pathological T stage.

Characteristics	Univariate OR (95% CI)	P-value	Multivariate OR (95% CI)	P-value
Sex	0.554 (0.157–1.952)	0.358		
Age	1.036 (0.982–1.093)	0.198		
Tumor size	1.281 (0.950–1.727)	0.105		
^**18**^**F-FDG PET**				
SUVmax	1.588 (1.140–2.212)	0.006	1.118 (0.745–1.680)	0.590
MTV	1.045 (1.014–1.078)	0.005	1.034 (1.003–1.065)	0.032
ISUP grade	36.667 (3.899–344.837)	0.002	12.859 (0.671–246.375)	0.197

## Discussion

The liver and kidneys are the two primary organs involved in gluconeogenesis. Interestingly, well-differentiated HCCs and RCCs are known to show low ^18^F-FDG uptake on PET/CT. Although the underlying molecular mechanisms for low ^18^F-FDG uptake in low-grade RCCs are poorly understood, they are attributable to preservation of the gluconeogenic properties of the kidneys with the expression of fructose 1,6-bisphosphatase 1^19^. The same mechanism has also been reported in low-grade HCCs with low ^18^F-FDG uptake^20^. Many studies have investigated the clinical value of ^18^F-FDG uptake before surgery for HCCs. For example, preoperative SUV_max_, which is reflective of tumour differentiation, was useful in selecting patients who would benefit from major hepatectomy rather than minor hepatectomy^10^. Preoperative ^18^F-FDG uptake is a significant imaging biomarker of microvascular invasion^21^. In a multicentre retrospective cohort study, preoperative ^18^F-FDG uptake was a prognostic factor for overall survival in patients with Barcelona clinic liver cancer (BCLC) stage 0 or A^22^. In addition, preoperative MTV was predictive of recurrence-free survival after curative resection^23^.

Unlike in HCCs, the predictive or prognostic value of variable ^18^F-FDG uptake remains to be elucidated in RCCs. In a recent report, patients with clear cell RCCs with a preoperatively elevated tumour to liver SUV ratio (TLR) on ^18^F-FDG PET/CT had significantly unfavourable survival outcomes after nephrectomy^17^. Of SUV_max_ and TLR on ^18^F-FDG PET/CT, primary tumour size, pTNM stage, WHO/ISUP grade, venous tumour thrombus, and adjuvant therapy, pTNM stage and TLR were independent prognostic predictors for disease-free survival. Therefore, close monitoring of patients with high TLR and pTNM stages is recommended to detect disease progression as early as possible. Instead of predicting patient outcomes, our prospective study focused on the use of ^18^F-FDG PET/CT for local tumour staging because it has an impact on deciding the best type of surgery for patients. We found that MTV, a volumetric parameter of metabolically active tumours, but not SUV_max_, as a unidimensional measure of ^18^F-FDG uptake on PET/CT, was a significant predictive factor for extended T stages (≥ pT3a) in patients with RCCs at staging. There are strengths and limitations of MTV^24–26^. Despite MTV having significant prognostic value for patient survival, there are limitations in measuring MTV due to a lack of standardized methods. Different methods are proposed and a wide range of threshold levels have been used to calculate the volume based PET/CT parameters. Further studies are needed to see whether different methods of measuring MTV would confirm the value of MTV in predicting extended T stages in RCC patients.

Regardless of tumour size, tumours can be staged as pT3a due to renal vein invasion, renal sinus invasion, or extracapsular extension, which may be microscopic and, thus, not readily appreciated on preoperative CT examination^5,27,28^. In this study, we evaluated metabolic parameters including SUV_max_ and MTV on ^18^F-FDG PET/CT and clinicopathological factors to predict extended pT stages. In the univariate analysis, metabolic imaging parameters and ISUP grade, but not tumour size, were significant predictive factors for the extended group. This result was consistent with the current AJCC staging system, in which tumour size is important only in further categorising tumours in the limited T stage^29^. In the multivariate analysis, MTV was the only significant predictor for extended pT stages, and a cut-off MTV of 21.2 showed a high AUC of 0.944 (95% CI, 0.818–0.992; p < 0.001). Given the significantly lower AUC of 0.824 for cT stages, our study showed the potential value of metabolically active tumour volume in predicting biological tumour aggressiveness in patients with RCCs. Further studies are needed to determine the role of preoperative MTV in selecting patients who would benefit from radical nephrectomy rather than partial nephrectomy.

Previous studies have found that high ^18^F-FDG uptake in RCCs is associated with high ISUP grades^30^. In addition to ISUP grade, LVI has been added as a new prognostic factor for cancer-specific survival and metastasis-free survival in RCC cases^29^. To date, there have been no studies on the association between ^18^F-FDG uptake and LVI. In this study, tumour size and ^18^F-FDG metabolic parameters were compared with regard to ISUP grades and LVI on pathology. We found no correlation between tumour size and ISUP grade or SUV_max_ of the primary tumours. Unlike tumour size, both SUV_max_ and MTV were higher in high-grade RCCs than in low-grade RCCs; in particular, MTV was significantly higher in RCCs in the presence of LVI than in the absence of LVI. Our results suggest that the volume of glycolysis in RCCs could be an important ^18^F-FDG PET/CT parameter indicating aggressive tumour biology, such as ISUP grade and LVI.

In line with the previous results, we found that tumours with low ISUP grades tended to have low SUV_max_, posing a potential risk of missing low-grade tumours on ^18^F-FDG PET/CT. Therefore, if the detection of primary RCCs is of major concern, ^18^F-FDG PET/CT may not be the best modality for screening. However, it would be beneficial to use metabolic features on ^18^F-FDG PET/CT to provide information on aggressive tumour behaviour such as ISUP grade, LVI, and extended pT stages. Recently, deep neural networks have made it possible to detect cancer at extremely low effective radiation doses of 0.11 mSv^31^. Localised imaging of the kidneys could be performed using an ultralow dose of ^18^F-FDG using deep learning algorithms to simulate full-dose data. If positive uptake by the primary tumour is observed, a standard dose of ^18^F-FDG can be injected, and conventional whole-body imaging can be performed to evaluate potential extrarenal metastases. This protocol could maximise the usefulness of ^18^F-FDG PET/CT by reducing the cost and unnecessary radiation dose to patients whose primary tumour shows no increase in ^18^F-FDG uptake. Further studies are needed to determine whether low-dose regional ^18^F-FDG PET/CT assisted by deep learning could be beneficial in translating the unique metabolic signature of RCCs into clinical practice to determine the scope of surgery and predict patient outcomes.

Our study had several limitations. First, the sample size was relatively small (n = 38) and a low sample can lead a wider confidence interval in ROC curve analysis; however, this was a prospective study that involved drug-naïve patients and definite inclusion criteria to reduce selection bias. Second, most of the RCCs were of clear cell types, and the number of papillary and chromophobe types was very small. Given that there may be differences in ^18^F-FDG uptake depending on RCC subtypes, further research is needed for subtype-specific analysis. Third, cT1 tumours were not included in this study because the aim of this study was to predict extended pT stages.

## Conclusion

MTV on ^18^F-FDG PET/CT is the only predictive factor for extended pT stages (≥ pT3a) in patients with RCCs at staging. In addition, high MTV on PET/CT, but not tumour size, was significantly correlated with aggressive pathologic features such as ISUP grade and LVI. ^18^F-FDG PET/CT seems to be a promising imaging tool when accurate prediction of pT stages is critical to aid decisions regarding the best type of surgery.

## Data Availability

The datasets generated during and/or analysed during the current study are available from the corresponding author on reasonable request.
